# Neuroprotective properties of transition metal dichalcogenide nanoflowers alleviate acute and chronic neurological conditions linked to mitochondrial dysfunction

**DOI:** 10.1016/j.jbc.2025.108498

**Published:** 2025-04-09

**Authors:** Charles L. Mitchell, Mikhail Matveyenka, Dmitry Kurouski

**Affiliations:** 1 Interdisciplinary Program in Genetics and Genomics, Texas A&M University, College Station, Texas, USA; 2 Department of Biochemistry and Biophysics, Texas A&M University, College Station, Texas, USA

**Keywords:** molybdenum disulfide, molybdenum diselenide, neurons, ROS, mitochondria

## Abstract

Mitochondrial dysfunction is an expected cause of etiology and progression in numerous human neurological pathologies, including stroke, Alzheimer's, and Parkinson's diseases. Therefore, a neuroprotective treatment is an urgent and unmet need. Transition metal dichalcogenide nanoflowers (TMD NFs) exhibit unique biological properties. However, neuroprotective properties of these nanomaterials remain poorly understood. In the current study, the biological effect of molybdenum disulfide and molybdenum diselenide TMD NFs on neurons and astrocytes was investigated. It was found that both nanomaterials lowered reactive oxygen species levels, reduced mitochondrial impairment, and increased mitochondrial biogenesis. Neuroprotective effects of both TMD NFs resulted from upregulation of the peroxisome proliferator-activated receptor gamma coactivator 1 alpha pathway, the biological system responsible for mitochondrial biogenesis. Furthermore, administration of TMD NFs to *Caenorhabditis elegans* extended lifespan of the nematodes. These results indicate that TMD NFs can be used as novel neuroprotective therapeutic agents against acute and chronic neurological condition linked to mitochondrial dysfunction.

Neurological conditions are pathologies that affect central and peripheral nervous systems. There are over 600 known neurological diseases that can be broadly categorized into three subtypes: neurotraumatic, neurodegenerative, and neuropsychiatric diseases (1, 2). Neurological conditions exhibit excitotoxicity, oxidative stress, and neuroinflammation linked to irreversible changes in mitochondrial homeostasis (2, 3, 4). Mitochondria have long been direct targets for therapeutic treatments, *via* targeting mitochondria’s role in apoptosis in cancer (5), reducing reactive oxygen species in neurodegenerative diseases (6, 7), and achieving and stabilizing normal physiological function in direct mitochondrial diseases, like Barth syndrome (8) and primary mitochondrial myopathies (9). Researchers target the mitochondria under the guise that by balancing mitochondria metabolism and mitophagy, maintaining calcium ion (Ca^2+^) concentration within the mitochondria, and regulating mitochondrial dynamics, these therapeutics can create an antiaging and neuroprotective effect in the patient (10). Certain genes responsible for mitochondrial biogenesis (peroxisome proliferator-activated receptor gamma coactivator 1 alpha [PGC-1α]) (11, 12, 13), mitochondrial dynamics (14, 15), and calcium homeostasis (IP3R) (16, 17) have been privy to multiple studies. While there are many ongoing studies for novel therapeutics, no current therapeutic treatment achieves the unmet need of neuroprotection (18).

Transition metal dichalcogenide nanoflowers (TMD NFs), including molybdenum disulfide (MoS_2_) and molybdenum diselenide (MoSe_2_), are novel nanomaterials that exhibit high surface-to-volume ratio *via* self-organization of individual sheets into higher coordinated structures (19). Although optical, electronic, and photocatalytic properties of TMD NFs are well characterized, biological activity of these nanostructures remains poorly understood. Several pieces of evidence indicate that TMD NFs showed promising therapeutic effects in cancer biology and neurodegenerative diseases (20, 21).

In the current study, the extent to which MoS_2_ and MoSe_2_ could enact neuroprotective properties to neurons and astrocytes was studied. Our results showed TMD NFs reduced mitochondrial impairment *via* suppression of reactive oxygen species (ROS) levels *in vitro*. Furthermore, administration of TMD NFs to *Caenorhabditis elegans* extended lifespan of the nematodes.

## Results and discussion

**Figure 1:**
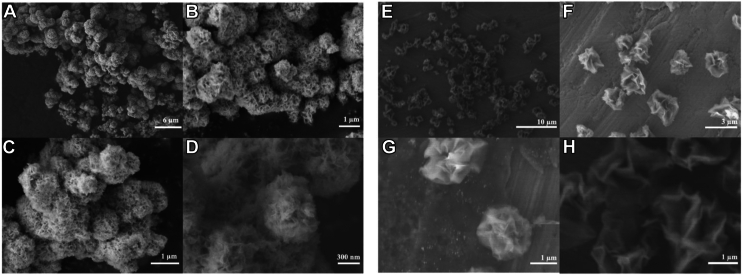
**SEM images of both MoS_2_ (*left*) and MoSe_2_ (*right*) nanoflowers.** The magnification of each image increases from 3000 X (*A*) to 50,000 X (*D*) for MoS_2_, and from 2700 X (*E*) to 20,000 X (*H*) for MoSe_2_. Each NF type displays the characteristic ridged surface that lends to a very high surface area-to-volume ratio. MoS_2_, molybdenum disulfide; MoSe_2_, molybdenum diselenide; SEM, scanning electron microscope.

MoS_2_ and MoSe_2_ TMDs were synthesized using hydrothermal synthesis in teflon-lined autoclaves. Scanning electron microscope revealed that both TMDs were assembled into dense NF-like structures with characteristically high surface area-to-volume ratio, with MoS_2_ measured between 300 and 600 nm and MoSe_2_ less than ∼1 μm (Fig. 1).

**Figure 2:**
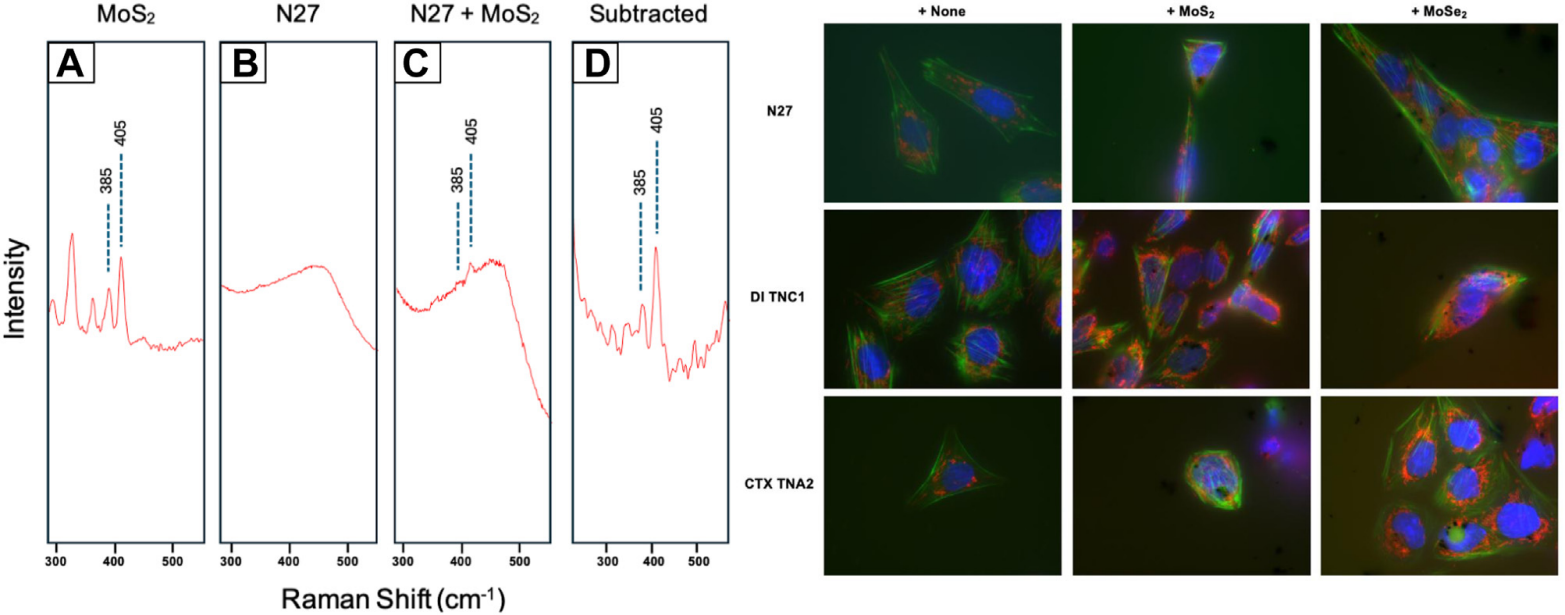
**Cellular uptake of TMD NFs into neurons and astrocytes.***Left*: Collected Raman spectra in the 300 to 500 wavenumber region for (*A*) powdered MoS_2_ NFs, (*B*) plated control N27 neurons, (*C*) plated N27 neurons treated with MoS_2_ NFs, and (*D*) the subtracted spectra from (*B* and *C*). *Right:* Fluorescent cell imaging of rat N27 neurons (*top row*), DI TNC1 astrocytes (*middle row*), and CTX TNA2 astrocytes (*bottom row*) when administered no NFs (*first column*), MoS_2_ NFs (*middle column*), or MoSe_2_ NFs (*last column*). MoS_2_, molybdenum disulfide; MoSe_2_, molybdenum diselenide; NF, nanoflower.

Cellular uptake of NFs was confirmed *via* both Raman spectroscopy and fluorescent imaging. Cells were treated with 10% solutions of respective concentrations of MoS_2_ and MoSe_2_ and allowed to incubate for 24 h. Raman spectra were acquired with a 532 nm laser from individual cells exposed to NFs and the dry NFs material. Raman spectra of dry MoS_2_ exhibited two peaks centered at 385 and 405 cm^-1^ that correspond to E_2g_ and A_1g_ vibrations of MoS_2_ (22), (Fig. 2). The same vibrational bands were observed in Raman spectra acquired from cells exposed to MoS_2_. These results indicate that analyzed cells possessed MoS_2_ NFs. Fluorescent imaging confirmed the presence of NFs in N27 neurons, DI astrocytes, and CTX astrocytes, (Fig. 2).

**Figure 3:**
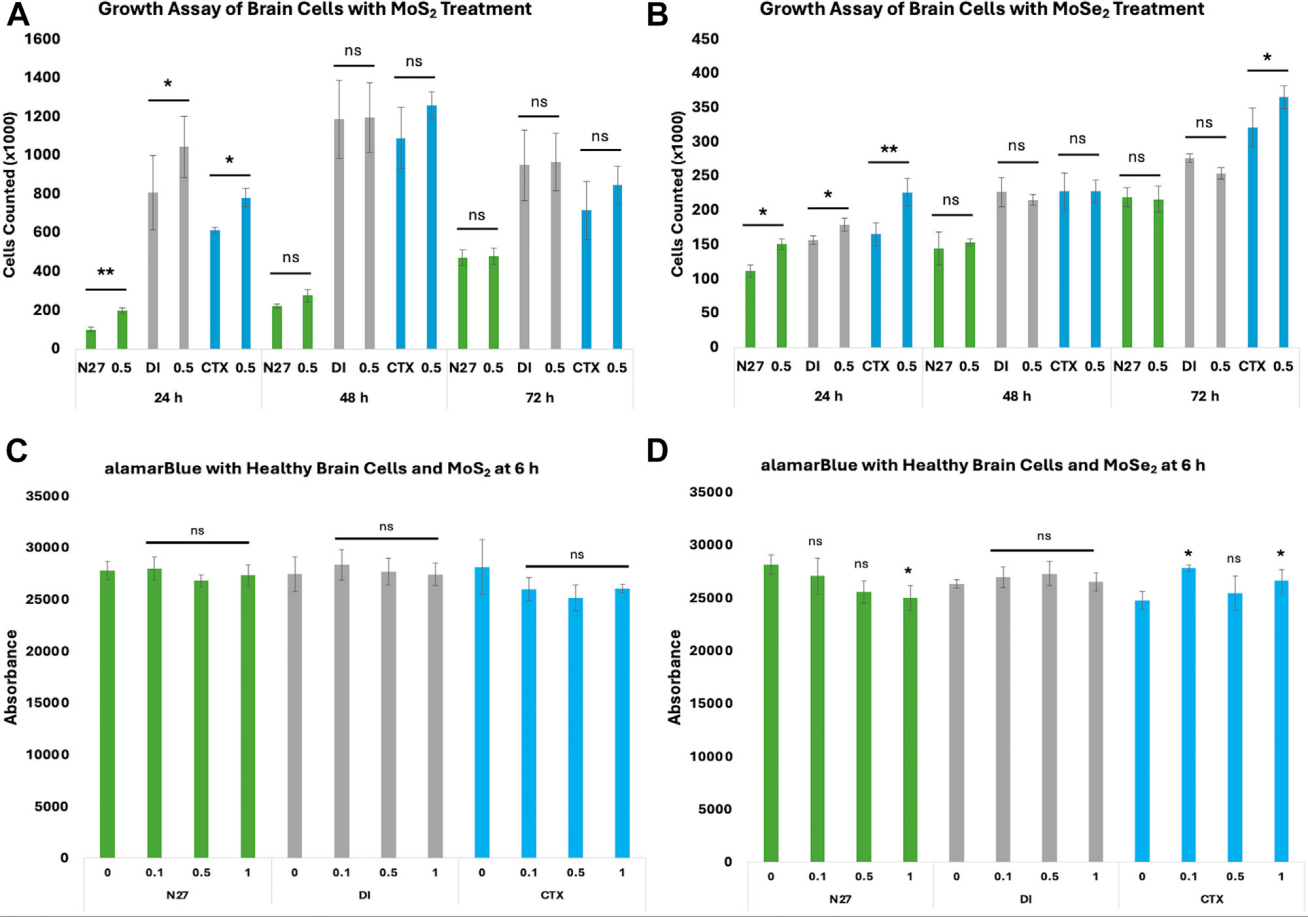
**Effects of TMDs on cellular proliferation and cell growth.** Growth curve assays of healthy rat brain cell types treated with MoS_2_ (*A*) and MoSe_2_ (*B*). Rat N27 neurons (*green*), rat DI TNC1 (*gray*), and rat CTX TNA2 (*blue*) were all treated with solutions of 0.5 mg/ml NFs and counted after 24, 48, and 72 h. Proliferation assay utilizing alamarBlue reagent to determine the proliferation of all tested cell types when treated for 6 h with MoS_2_ (*C*) and MoSe_2_ (*D*). Rat N27 neurons (*green*), rat DI TNC1 (*gray*), and rat CTX TNA2 (*blue*) were all treated with solutions of 0.1 mg/ml, 0.5 mg/ml, or 1.0 mg/ml of NFs then received alamarBlue for testing. MoS_2_, molybdenum disulfide; MoSe_2_, molybdenum diselenide; NF, nanoflower.

Cellular growth curve assays determined the extent to which TMD NFs altered cell proliferation. The number of N27, DI, and CTX cells was determined at 24 h, 48 h, and 72 h posttreatment with TMDs. N27, DI, and CTX cells treated with MoS_2_ experienced a significant boost in cell count within the first 24 h, (Fig. 3). Specifically, rat N27 neurons exhibited a 93.2% increase in cell number, while both the rat DI TNC1 and CTX TNA2 astrocytes showed a smaller increase in the rate of cell proliferation, 29.2% and 27.4%, respectively. Although all cell types initially underwent a significant increase in the cell count within the first 24 h, no significant changes in the rate of cell proliferation were observed at the following 48 h and 72 h. These results indicated that MoSe_2_ strongly enhanced the cell proliferation only within the first 24 h after their introduction to N27, DI, and CTX cells.

Similarly, N27, DI, and CTX cells treated with MoSe_2_ experienced a significant initial increase in cell count, (Fig. 3). N27 neurons exhibited a 34.7%, while DI TNC1 astrocytes demonstrated a 14.2% increase in total counted cell number within the first 24 h. Cellular growth curve assay revealed the highest boost (36.9% increase) in the proliferation of CTX TNA2 astrocytes after the first 24 h of cell exposure. Similarly to MoS_2_, none of the tested cell types experienced a significant difference in total cell number after 48 h of their exposure to MoSe_2_ TMDs. After 72 h, a significant increase in total number of cells was observed only for CTX astrocytes, whereas no significant changes in the cell counts were evident for N27 and DI cells. These results indicated that both neurons and astrocytes exhibited similar increases in cell proliferation within the first 24 h after addition of MoS_2_ and MoSe_2_ to the cell media. However, observed differences in the cell proliferation of CTX astrocytes exposed to MoS_2_ and MoSe_2_ at 24 h and 72 h stages suggests the magnitude of cell proliferation was linked to the chemical nature of TMDs.

An alamarBlue assay was used to assess changes in the proliferation of N27, DI, and CTX cells after 6 h of after administration of both MoS_2_ and MoSe_2_ (Fig. 3). AlamarBlue revealed no significant changes in the reducing potential of N27, DI, and CTX cells exposed to MoS_2_ compared to control cells. This result indicated that MoS_2_ NFs did not exhibit any detrimental effects on the brain cells. On average, N27, DI, and CTX cells duplicate every 12 h. Consequently, our results also indicate that MoS_2_ enhance viability and, consequently, property to replicate between 6 and 12 h after cell exposition to TMDs.

Similarly to MoS_2_, neuronal cells exposed to low concentrations of MoSe_2_ showed no changes in the reducing potential within the first 6 h (Fig. 3). However, at 1.0 mg/ml concentration of MoSe_2_, a decrease in 11.2% of the cell reducing potential was observed. These results indicate that high concentration of MoSe_2_ can cause slight detrimental effects on cell proliferation potential. These effects were not evidence for DI astrocytes. At the same time, CTX astrocytes positively responded on MoSe_2_ treatment exhibiting an increase in the reducing potential. These results demonstrate that different cell types exhibit slightly different responses on the treatment with TMDs.

**Figure 4:**
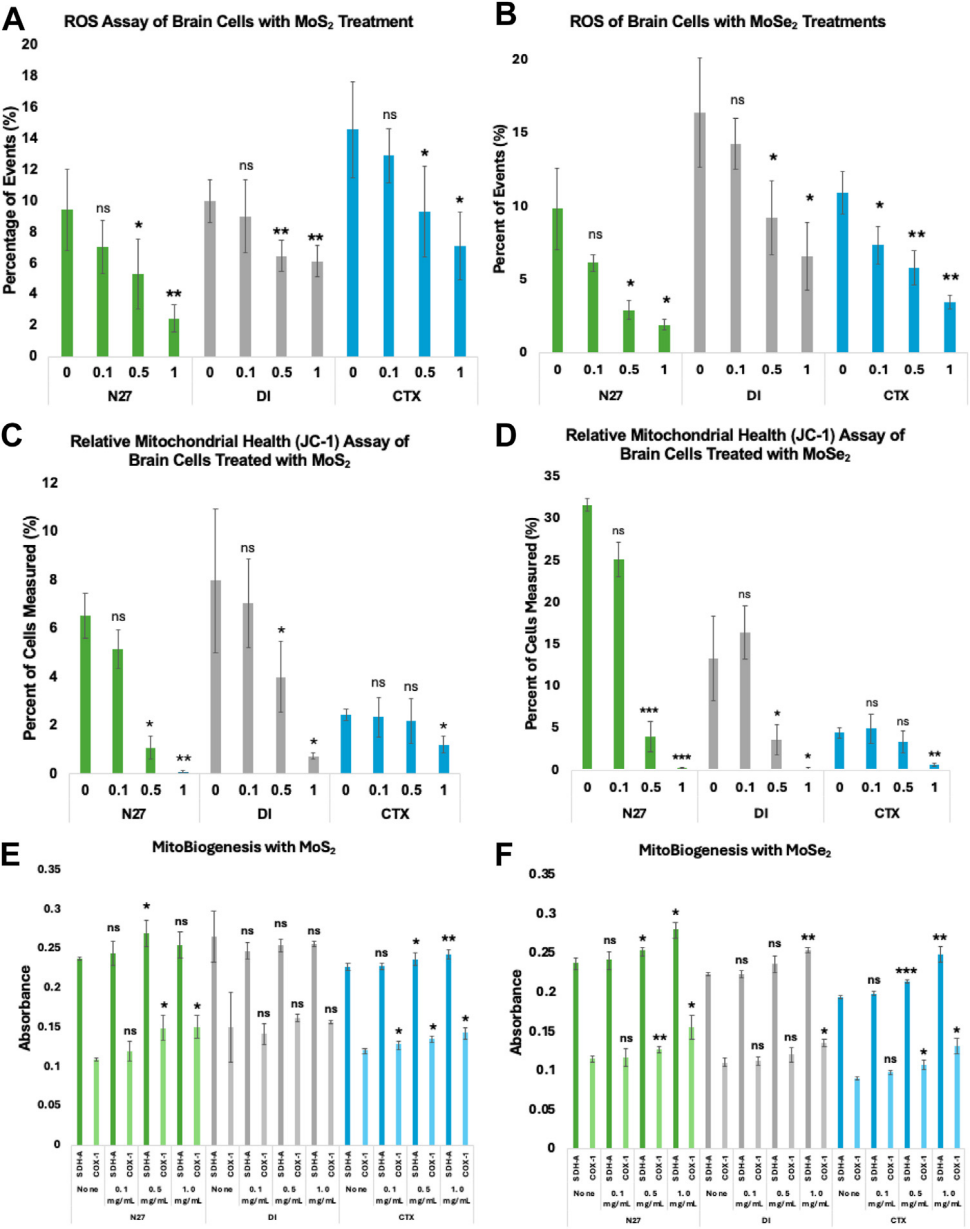
**Mitochondrial effects induced by TMD NFs in neurons and astrocytes.** Reactive oxygen species assay using CellROX reagent to understand the effects that MoS_2_ (*A*) and MoSe_2_ (*B*) nanoflowers have on the ROS of N27 neurons (*green*), DI astrocytes (*gray*), and CTX astrocytes (*blue*). Relative mitochondrial health assay using JC-1 to measure the percent of green monomer units of the dye in N27, DI, and CTX cells after treatment with MoS_2_ (*C*) and MoSe_2_ (*D*). Performed ELISA for the succinate dehydrogenase (SDH-A) and cytochrome c oxidase 1 (COX-1) proteins to quantify the mitochondrial proteins present as a result of TMD NF treatments. The graphs depict the absorbance measured from the ELISA for SDH-A (*darker color*) and COX-1 (*lighter color*) for N27 neurons, DI astrocytes, and CTX astrocytes when treated with MoS_2_ (*E*) and MoSe_2_ (*F*). MoS_2_, molybdenum disulfide; MoSe_2_, molybdenum diselenide; TMD, transition metal dichalcogenide.

Cells that received MoS_2_ treatments exhibited substantially lower ROS levels than the control, (Fig. 4). Specifically, N27 neurons exposed to 0.1 mg/ml of MoS_2_ demonstrated a 25.8% decrease in ROS levels. As the concentration of MoS_2_ in the cell media increased to 0.5 and 1.0 mg/ml, there was a reciprocal decrease of ROS in N27 neurons by 43.8% and 74.2%, respectively. In DI astrocytes, a 9.7% decrease in ROS was observed for the lowest concentration of MoS_2_. The cells treated with 0.5 and 1.0 mg/ml of MoS_2_ exhibited a greater magnitude of the decrease in the ROS levels (35.5% and 38.8%, accordingly). CTX astrocytes treated with 0.1 mg/ml MoS_2_ experienced an 11.4% decrease in the ROS, whereas the cells treated with 0.5 mg/ml and 1.0 mg/ml of MoS_2_ had 36.2% and 51.3% lower ROS levels than the control.

Cells treated with MoSe_2_ experienced a suppressed production of (Fig. 4). Compared to other cell types, N27 cells experienced the strongest suppression of ROS levels. When treated with 0.1 mg/ml of MoSe_2_, N27 neurons exhibited 37.5% decrease in ROS compared to the control. Furthermore, 70.3% and 80.4% suppression in ROS levels was observed in neurons treated with 0.5 and 1.0 mg/ml MoSe_2_, respectively. In DI astrocytes, the trend of decreasing ROS was observed. When treated with 0.1 mg/ml MoSe_2_, there was a 12.9% decrease in measured ROS. As the concentration of administered NFs increased to 0.5 and 1.0 mg/ml, the cells demonstrated 43.8% and 59.8% decreases in ROS levels, respectively. After receiving the lowest dose of MoSe_2_, the CTX cells experienced a reported 32.6% decrease in overall ROS. As the dosage concentration increased to 0.5 and 1.0 mg/ml MoSe_2_, the cells showed significant 46.6% and 67.9% decreases in ROS, respectively.

The extent of mitochondrial impairment in neurons and astrocytes was measured using JC-1. Administration of MoS_2_ treatment lowered mitochondrial impairment in N27 neurons, (Fig. 4). Cells exposed to 0.1 mg/ml of MoS_2_ exhibited 21.4% lower damage of cell mitochondria than the control. JC-1 assays revealed that 83.7% and 98.9%, suppression of mitochondrial impairment was observed in N27 neurons exposed to 0.5 mg/ml and 1.0 mg/ml of MoS_2_, respectively. Thus, MoS_2_ helped to decelerate the progression of naturally occurring mitochondrial damage. In DI astrocytes, the lowest concentration (0.1 mg/ml) resulted in 11.7% decrease in mitochondrial impairment. DI astrocytes treated with 0.5 mg/ml of MoS_2_ experienced a 49.8% decrease in the mitochondrial damage, whereas a 90.8% decrease in the organelle impairment was observed for DI astrocytes treated with 1.0 mg/ml of MoS_2_. CTX astrocytes treated with 0.1 and 0.5 mg/ml exhibited much lower magnitude of mitochondrial survival equal to 4.1% and 10.9%, respectively. At the highest concentration of MoS_2_ (1.0 mg/ml), however, there was a significant 50.7% decrease in mitochondrial impairment in CTX astrocytes.

Similarly, N27 neurons that received MoSe_2_ treatments exhibited 20.6% increase in the mitochondrial survival compared to the control, whereas cell exposed to 0.5 and 1.0 mg/ml of MoSe_2_ demonstrated 87.3% and 99.1% suppression of mitochondrial damage, (Fig. 4). DI astrocytes did not exhibit a significant change in the mitochondrial impairment when treated with the lowest concentration of MoSe_2_, but a significant decrease in the mitochondrial damage (72.8% and 98.5%, respectively) was observed in DI astrocytes treated with 0.5 and 1.0 mg/ml of MoSe_2_. CTX astrocytes did not exhibit any significant changes when treated with 0.1 and 0.5 mg/ml of MoSe_2_. However, 85.2% increase in the suppression of mitochondrial impairment in CTX astrocytes treated with 1.0 mg/ml of MoSe_2_.

The experiments described above thus far have shown the phenotypic effects that TMD NF treatments induce. To better elucidate the mechanistic effects resulting from TMD NF exposure, enzyme-linked immunosorbent assays, and quantitative PCRs (qPCRs) were conducted.

The utilized in-cell ELISA detects treatment-induced effects on mitochondrial biogenesis *via* measurement of two mitochondrial proteins: nuclear encoded succinate dehydrogenase subunit A (SDH-A) and mitochondrial encoded cytochrome c oxidase subunit 1 (COX-I). N27 neurons experienced an increase in both SDH-A and COX-I levels for all tested concentrations of MoS_2_ NFs, (Fig. 4). Treatments with 0.1 mg/ml MoS_2_ yielded the smallest increase in both SDH-A and COX-I, with only a 2.9% and 9.8% increase in the respective protein levels. The higher concentration treatments induced much higher increases, with the 0.5 mg/ml resulting in 13.4% and 37.0% increases in SDH-A and COX-I, while the 1.0 mg/ml treatment gave rise to 7.2% and 38.2% increases, respectively. The DI astrocytes that received MoS_2_ treatments did not exhibit any significant changes in SDH-A or COX-I expression levels. The CTX astrocytes received MoS_2_ experienced increases in both SDH-A and COX-I levels at every test concentration, similarly to the N27 neurons. At the 0.1 mg/ml concentration, there were minor increases of 0.5% and 6.6% in SDH-A and COX-I respectively. As the treatment concentrations increased, there were significant increases of 4.3% and 12.4% respectively for the 0.5 mg/ml group, and significant 7.2% and 19.1%, respectively, increased for the 1.0 mg/ml group.

When treated with MoSe_2_ NFs, N27 experienced a dosage dependent increase in both SDH-A and COX-I levels, (Fig. 4). At the lowest concentration of 0.1 mg/ml, there was a measured increase of 1.9% and 1.4% in SDH-A and COX-I respectively. As the concentration increased to 0.5 mg/ml, there was significant increases of 6.5% and 10.1% of the respective proteins. At the highest concentration of 1.0 mg/ml, there was the highest significant increases of 18.2% and 35.1%, respectively. The DI astrocytes treated with MoSe_2_ also experienced a dose-dependent increase of SDH-A and COX-I levels, although not as significantly as N27 neurons. At the lowest concentration of 0.1 mg/ml, there was only slight 0.3% and 1.5% respective increases in SDH-A and COX-I. When treated with the 0.5 mg/ml concentration, there were 5.8% and 9.1% increases in the respective protein levels. The only significant increases were measured when treated with 1.0 mg/ml MoSe_2_, with a positive change of 14.0% and 22.6%, respectively. The measured effects of MoSe_2_ were greater in CTX astrocytes over the MoS_2_ treatments. At the 0.1 mg/ml concentration, there was a slight increase of 2.5% and 7.7% in SDH-A and COX-I, respectively. At the 0.5 mg/ml concentration, there were significant increases of 10.5% and 18.2%, respectively. At the highest concentration of 1.0 mg/ml, the significant increases in protein levels were measured at 28.5% and 44.6%, respectively.

**Figure 5:**
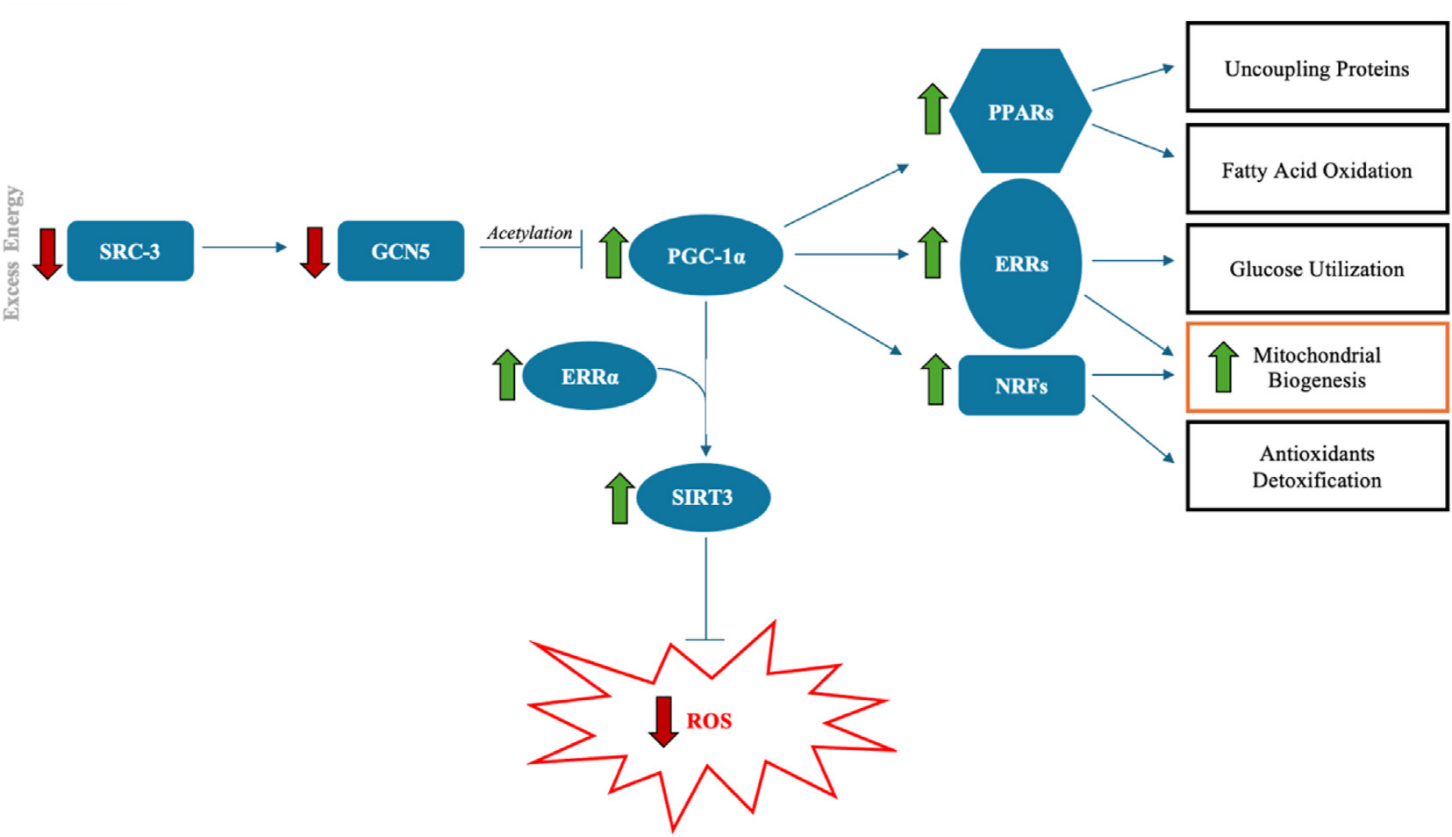
**Diagram of the PGC-1α biological pathway with important core components shown.** Custom qPCR primers were generated for all components *shaded blue*. *Green arrows* indicate measured upregulation, while *red arrows* indicate measured downregulation. PGC-1α, peroxisome proliferator-activated receptor gamma coactivator 1 alpha; qPCR, quantitative PCR.

Recently reported studies by the Gaharwar group (23) showed that MoS_2_ NFs exhibiting different atomic vacancies triggered upregulation of PGC-1α. PGC-1α pathway is directly responsible for mitochondrial biogenesis in the cell and thus, plays key roles in other processes such as ROS regulation, glucose utilization, fatty acid oxidation, and antioxidant detoxification, (Fig. 5). Therefore, qPCR was utilized to quantify changes in the expression of proteins in this pathway when exposed to both MoS_2_ and MoSe_2_ NFs.

The central component of the pathway, PGC-1α, is regulated by the sirtuin (SIRT)1 protein. The Gaharwar group reported an upregulation of SIRT1 and PGC-1α expression in response to MoS_2_ administration (23). In another upstream arm of the pathway, steroid receptor coactivator-3 (SRC-3) monitors energy excess and acts as a coactivator of nuclear receptors and transcription factors (24). SRC-3 is positive feedback regulator of general control nondepressible 5 (GCN5) that inhibits PGC-1α and downregulates its expression through acetylation (25). Both SRC-3 and GCN5 were both downregulated when administered MoS_2_ and MoSe_2_ NFs, with MoS_2_ resulting in a greater downregulation in both genes. SRC-3 only had 36.0% and 81.3% expression after exposure to MoS_2_ and MoSe_2_. GCN5 expression was reduced to 76.5% with MoS_2_ and did not change in response to MoSe_2_.

PGC-1α suppresses ROS activity by activation of SIRT3 and estrogen-related receptor alpha (ERRα) (26). Our results showed that MoSe_2_ suppressed ROS more efficiently than MoS_2_ NFs in neurons. Indeed, qPCR revealed that SIRT3 was 5% and 31% upregulated in cells exposed to MoS_2_ and MoSe_2_ NFs, respectively. Consequently, ERRα experienced an 89% and 7.8-fold upregulation when administer MoS_2_ and MoSe_2_ NFs.

PGC-1α regulates multiple cellular processes, such as uncoupling proteins, fatty acid oxidation, glucose utilization, antioxidants detoxification, and mitochondrial biogenesis (26, 27) *via* peroxisome proliferator-activated receptor alpha, gamma, and delta (PPAR α/γ/δ), ERR α/β/γ, and nuclear respiratory factor 1 and 2 (NRF 1/2). qPCR revealed that in neurons exposed to TMD NFs, the expression of these protein groups was strongly upregulated to varying magnitudes. Analysis of qPCR for PPARα and PPARδ showed that MoSe_2_ triggered a greater upregulation of these genes *versus* MoS_2_. Specifically, PPARα experienced a 3.9-fold and 3.4-fold, respectively, while PPARδ 2.2-fold and 27% increases. PPARγ had the inverse effect, with MoS_2_ causing a higher upregulation than MoSe_2_. Analysis revealed 6.2-fold and 3.9-fold increases in expression, respectively. As previously stated, ERRα experienced a higher upregulation in response to MoSe_2_ over MoS_2_. Contrarily, ERRβ experienced a higher upregulation when administered MoS_2_ than MoSe_2_, with 74.6% and 40.1% measured increases. Lastly, investigation into changes in the expression of NRF2 was conducted. When administered MoS_2_, NRF2 had a modest upregulation of only 7.5%, but had a steeper upregulation of 51.9% when treated with MoSe_2_.

*C. elegans* are a well-established, multicellular model organism (28) broadly utilized to investigate neurological effects and toxicity of molecular analytes (29, 30), tracking developmental stages of neuronal networks in embryos (31), as well as molecular mechanisms of neurodegenerative diseases (32). A WT N2 *C. elegans* strain was used to investigate the extent to which TMD NFs on could alter the lifespan of the worms.

**Figure 6:**
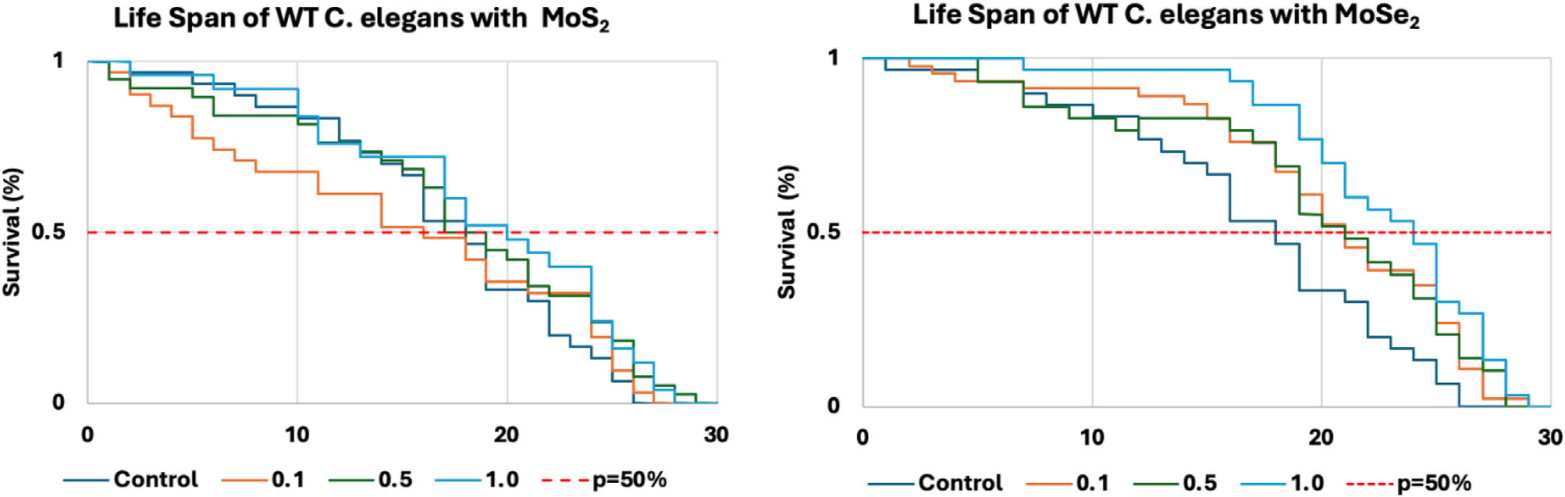
**Calculated Kaplan–Meier life expectancy curves of *C. elegans* that were administered different concentrations of MoS_2_ NFs (*left*) and MoSe_2_ NFs (*right*).** MoS_2_, molybdenum disulfide; MoSe_2_, molybdenum diselenide; NF, nanoflower.

The *C. elegans* that received MoS_2_ supplementation did not experience a great shift in survival expectancy (*p* = 50%). The MoS_2_ supplemented worms had a calculated survival expectancy of 18 ± 2 days, as shown in (Fig. 6). The 0.1 mg/ml MoS_2_ group had a slightly lower *p* = 50% at 16 days. As the concentration of MoS_2_ increased, the 0.5 mg/ml and 1.0 mg/ml groups experienced higher *p* = 50% values of 19 days and 20 days, respectively. These two groups exceeded the survival expectancy of the healthy control worms, which experienced a survival expectancy of 18 days.

The *C. elegans* that received MoSe_2_ supplementation experienced a much greater shift in survival expectancy. The MoSe_2_ supplemented worms had a calculated survival expectancy of 22 ± 1.7 days, as shown in (Fig. 6). All concentrations of MoSe_2_ supplementation resulted in an increase in survival expectancy over the healthy control groups. Both the 0.1 mg/ml and 0.5 mg/ml had a calculated *p* = 50% value of 21 days, 3 days higher than the control. The group that experienced the greatest change in *p* = 50% was the 1.0 mg/ml MoSe_2_ worms, with a survival expectancy of 24 days. This represents an increase of 6 days over the healthy WT *C. elegans*. Furthermore, we observed very low mortality during the first 15 days for *C. elegans* exposed to 0.5 and 1.0 mg/ml MoSe_2_. These results demonstrate that MoSe_2_ drastically extends the lifespan of *C. elegans* and strongly reduces nematode death during the first half of their life.

Neurological conditions are the second leading cause of death and are the leading cause of disability worldwide (33). Proper mitochondrial function is vital for cellular homeostasis. In a dysfunctional state, mitochondria cause deleterious effects on the cell—including oxidative stress, secondary excitotoxicity, and insufficient production of ATP—which in turn can trigger apoptosis (34). No current treatment provides neuroprotective properties, leaving a large unmet need for a therapeutic targeting the mitochondria.

To meet this need, two structurally different NFs were synthesized. Cell biogenesis assay revealed that MoSe_2_ caused stronger biogenesis in neurons than MoS_2_. A strong decrease in the magnitude of mitochondrial impairment and ROS levels was observed in all cells exposed to MoS_2_ and MoSe_2_ NFs. The increase in mitochondrial biogenesis and *C. elegans* lifespan hint at TMD NFs’ neuroprotective potential. The magnitude of beneficial effects varies between NFs, and this observation further demonstrates that neuroprotective effects of TMD NFs are determined by their structure. Consequently, it becomes critically important to examine which characteristics of TMD NFs are the key player in biological and neuroprotective activity, with further research elucidating how size, morphology, and chemical composition affect how neuronal cells behave upon exposure. We hypothesize the size and surface area-to-volume ratio are the main driving characteristics to the tested TMD NFs’ neuroprotective potential. By manipulating key aspects of synthesis protocols, the neuroprotective activity of larger and smaller size NFs, TMD nanorods, and other dichalcogenides, including tungsten disulfide and tungsten diselenide, as well as TMD NF oxides remains of interest in planned future studies.

## Experimental procedures

### Materials

All chemicals received were utilized without further purification. MilliQ water was utilized in the synthesis of nanomaterials. Ammonium molybdate tetrahydrate ((NH_4_)_6_Mo_7_O_24_) 4H_2_O), thiourea 99% (NH_2_CSNH_2_), selenium powder, and sodium borohydride (NaBH_4_) were purchased from Sigma-Aldrich (Sigma-Aldrich).

### Synthesis of NFs

MoS_2_ NFs were synthesized using a hydrothermal synthesis reaction. For this, 50 ml solution of 0.01 mol (NH_4_)_6_Mo_7_O_24_ and 0.06 mol NH_2_CSNH_2_ in MilliQ water was kept for 30 min at room temperature prior to its transfer into 50-ml teflon-lined autoclave reactors. The autoclaves were exposed to 200 °C for 24 h. After autoclave reactors were allowed to cool to room temperature overnight, the resulting black precipitate was collected and centrifuged at 7000 rpm for 5 min. The samples were washed with MilliQ water and centrifuged twice at 7000 rpm for 5 min. Next, samples were washed with ethanol, placed on a glass Petri dish, and kept overnight in a dry hot air oven at 60 °C. The desiccated NFs were collected from the Petri dish and stored.

MoSe_2_ NFs were synthesized using a hydrothermal synthesis reaction. (NH_4_)_6_Mo_7_O_24_ (0.003 mol) and NaBH_4_ (0.1 g) were dissolved in 50 ml solution of MilliQ water and pure ethanol at a 1:1 ratio. Separately, 0.006 mol of selenium powder was dissolved in 10 ml of NaBH_4_, added to the solution of (NH_4_)_6_Mo_7_O_24_, and stirred for 30 min at room temperature. The obtained solution was transferred into two 50-ml teflon-lined autoclave reactors and placed at 200 °C for 48 h. After the heating was turned off, the reactor was allowed to cool to room temperature. The resulting black precipitate was collected and centrifuged at 7, 000 rpm for 5 min, washed with MilliQ water and centrifuged three times. The washed nanoparticles were placed on a glass Petri dish and allowed to dry in a hot air oven at 60 °C overnight. The desiccated NFs were collected and stored.

### Scanning electron microscopy

MoS_2_ and MoSe_2_ NFs were imaged with a scanning electron microscope at the Material Characterization Facility at Texas A&M University [RRID: SCR_022202].

### Cell culture

Dopaminergic midbrain N27 rat neurons were purchased from American Type Culture Collection and grown in RPMI 1640 medium (Thermo Fisher Scientific) supplemented with 10% heat-inactivated fetal bovine serum (Invitrogen).

CTX TNA2 and DI TNC1 rat astrocyte cells were purchased from American Type Culture Collection and grown in Dulbecco's modified Eagle's medium supplemented with 10% heat inactivated fetal bovine serum (Invitrogen).

All cells were cultured in T-75 flasks purchased from (Thermo Fisher Scientific) and incubated at 37 °C in 5% CO_2_ environment until utilized in experiments. Cells were passaged at approximately 90% confluency. All cell types were used prior to the 10th passage to ensure reliable results.

### NF treatments

Cells were seeded based on the quantity necessary for the assay being performed (10,000–100,000 cells per well). The cells were allowed to fully adhere overnight. After adhering, 10% of the media was removed. A 2 mg/ml stock of the NF was made in PBS. The 2 mg/ml stock was probe sonicated for 30 s to suspend the NFs completely. The 2 mg/ml stock was then serially diluted into the three tested concentrations: 1.0 mg/ml, 0.5 mg/ml, and 0.1 mg/ml. The same volume of removed media was then replaced by an equivalent volume of the tested concentration of NFs. The control cells that did not receive any NFs got an equivalent volume of PBS. The final volume of NFs for each sample is 10% solution consistent across all cell types and prepared concentrations of NFs.

### Raman spectroscopy

Confirmation of cellular uptake of NFs was conducted using Raman spectroscopy. The Raman spectra were collected using a TE-2000U Nikon inverted confocal microscope equipped with a solid-state laser generated continuous wavelength 532 nm light. Electromagnetic radiation was directed toward the sample directed using a 50/50 beam splitter and focused by 20× Nikon objective. Scattered light was collected using the same objective and directed into an IsoPlane-320 spectrometer (Princeton Instruments) equipped with a 1200 groove/nm grating. Prior to entering the spectrograph, elastically scattered photons were removed using a long-pass filter (Semrock). The inelastically scattered photons were collected using PIX-400B CCD (Princeton Instruments).

For the measurements, 10,000 cells were seeded in glass-bottom 96-well plate and allowed to adhere overnight. NF treatments were administered as previously described, and cells were incubated for 24 h. After incubation, the 96-well plate was mounted on the confocal Raman spectroscope. Individual cells were selected and placed in the focal plane of the laser. The Raman spectra were collected using 10 s acquisition time and 6.85 mW of light. In parallel, reference Raman spectra were collected from dry NFs.

### Proliferation assay

Growth assays for each cell type with each NF were conducted. Cells were placed in a 24-well plate and allowed to adhere for 24 h. Cells then either received a 10% concentration of PBS as a negative control or a 10% concentration treatment of 0.5 mg/ml of MoS_2_ or MoSe_2_. Cells were counted after 24, 48, and 72 h with an Invitrogen Countess 3 Automated Cell Counter (Thermo Fisher Scientific) after NF treatment and compared.

A proliferation assay was also conducted using alamarBlue HS Cell Viability Reagent (Thermo Fisher Scientific). After allowing 20,000 cells to adhere to the plate overnight, each well was treated with either PBS or the specified NF at each concentration. After 2 h, the alamarBlue reagent was added to the wells. The color change was observed, and the fluorescence was measured after 6 h.

### Mitochondrial health assays

Cells were plated in 96-well plates and treated with NFs according to parameters previously detailed. To assess the levels of ROS present in the cells, the wells were treated with CellROX Deep Red Reagent (Thermo Fisher Scientific). The treated cells were scanned and measured using an LSRII BD flow cytometer (BD). The data were analyzed, and cell toxicity was calculated using LSRII software. To assess the health of mitochondria in the NF-treated cells, the wells were treated with MitoProbe JC-1 (Thermo Fisher Scientific). The treated cells were scanned and measured using an LSRII BD flow cytometer (BD). The data were analyzed, and cell toxicity was calculated using LSRII software.

### Enzyme-linked immunosorbent assay

Mitochondrial biogenesis was quantified using the MitoBiogenesis In-Cell ELISA Colorimetric Kit (Abcam). Cells were treated according to the NF parameters previously stated. After 24 h of treatment, the cells were fixed to the plate using BD Cytofix fixation buffer (BD Biosciences). The cells were then blocked, permeabilized, and treated with primary and secondary antibodies. The cells were then treated with AP development solution and absorbance was measured using a plate reader to quantify expression of SDH-A. The media was removed, and the cells were treated with horseradish peroxidase development solution and absorbance was measured using a plate reader to quantify expression of COX-1. Data were saved, collected, and analyzed for reporting.

### Polymerase chain reaction

PCR for genes with roles in the mitochondrial biogenesis pathway was conducted, as well as mitochondria copy number, using GAPDH as a housekeeping gene. All primers were designed using the known rat gene sequences in the NCBI database and created using the Integrated DNA Technologies custom oligonucleotide PCR primer generation software. All primers were ordered and received from Integrated DNA Technologies. Cells were treated according to treatments described above. The cells were harvested and centrifuged to form a pellet. The pellet was resuspended in TRIzol Reagent (Thermo Fisher Scientific) and chloroform (Avantor). The solution was centrifuged, and RNA was extracted from the aqueous phase. The RNA was then converted to complimentary DNA to be used in the PCR reaction. The utilized primers are listed in Table 1.

**Table 1:** qPCR Primers

Gene	Sense primer	Antisense primer
*SIRT3*	5′ GCTCATGGGTCCTTTGTATCA 3′	5′ CCTAGCTGGACCACATCTTC 3′
*ERRα*	5′ TGGCCGACAGAAGTACAA 3′	5′ AGAGTGACAGTGAGGAGAAG 3′
*ERRß*	5′ CCTTTGTCCATCCCTTTCTC 3′	5′ CCTGTGTGTGTCTCTTTGTG 3′
*NRF2*	5′ CGAAGGAGAGGGAAGAATAAAG 3′	5′AGTACTCACTGGGAGAGTAAGG 3′
*PPARα*	5′ TTGTGCATGGCTGAGAAG 3′	5′ CAGCATCCCGTCTTTGTT 3′
*PPARγ*	5′ GGCCTCCCTGATGAATAAAG 3′	5′ CGGTCTCCACTGAGAATAATG 3′
*PPARδ*	5′ CATTGCCGCCATCATTCT 3′	5′ GTCTCACTCTCCGTCTTCTT 3′
*GCN5*	5′ GGAGAATGTGTCAGAGGATGAG 3′	5′ CTCCAGCTTCCAGTAGTTAAGG 3′
*SRC3*	5′ GCAGGAGAGCAAGTACATAGAG 3′	5′ CGGTTCACCACAAACAGAAAG 3′
*GAPDH*	5′ GGCACAGTCAAGGCTGAGAATG 3′	5′ ATGGTGGTGAAGACGCCAGTA 3′

ERR, estrogen-related receptor; NRF2, nuclear respiratory factor 2; PPAR, peroxisome proliferator-activated receptor alpha; qPCR, quantitative PCR; SIRT3, sirtuin 3; SRC-3, steroid receptor coactivator-3.

### *C. elegans in vivo* modeling

WT N2 *C. elegans* strain was acquired as a kind gift from Dr Michael Polymenis, Texas A&M University. The *C. elegans* were raised on NGM plates seeded with OP50 *Escherichia coli* and maintained at a constant 20 °C until reaching an egg-producing age. Age synchronization was conducted by collecting all worms and eggs and bleaching the solution to remove all adult worms, according to Sutphin & Kaeberlein’s protocol (35). Synchronized worms were allowed to reach “day 1 adult” age before being transferred onto experimental plates. Ten individual worms were moved onto each conditioned agar plate. The conditioned plates were produced by plating *E. coli* supplemented with TMD NFs. NF supplementation was performed by mixing concentrated 2% stocks with 10× concentrated OP50 *E. coli* at a 1:1 ratio before plating, quickly drying, and UV irradiating. Survival of the *C. elegans* was determined by counting the number of alive and dead organisms on each plate until no surviving worms were counted and then calculated using the Kaplan–Meier equation.

### Fluorescent imaging

Fluorescent imaging was conducted in an EVOS M5000 microscope (Thermo Fisher Scientific). To image the cellular skeleton, CellMask Green Actin Tracking Stain and CellMask Deep Red Actin Tracking stains were utilized. To locate and image mitochondria, MitoTracker Orange CM-H2TMRos were used. The nuclei were stained using NucBlue Live Cell Stain ReadyProbe reagent. To enable visualization of ROS levels and the extent of mitochondrial impairment, CellROX Deep Red reagent and MitoProbe JC-1 reagent were used, respectively. All fluorescent stains and reagents were acquired from Thermo Fisher Scientific.

To image NFs in the cells, N27 neurons, DI astrocytes, and CTX astrocytes were captured when incubated without NFs, as well as with 0.5 mg/ml MoS_2_, and with 0.5 mg/ml MoSe_2_ using green channel.

## Conclusions

The reported results indicate that TMD NFs facilitate mitochondrial biogenesis which results in the reduction of ROS-induced mitochondrial damage in neurons and astrocytes. These effects are exerted by the upregulation of the PGC-1α pathway. Thus, MoS_2_ and MoSe_2_ NFs hold a powerful potential to be neuroprotective therapeutics in neurological conditions with mitochondrial dysfunction.

## Data availability

Data will be available upon the reasonable request from the authors.

## Conflict of interest

The authors declare that they have no conflicts of interest with the contents of this article.
